# Classification of dry and wet macular degeneration based on the ConvNeXT model

**DOI:** 10.3389/fncom.2022.1079155

**Published:** 2022-12-08

**Authors:** Maonian Wu, Ying Lu, Xiangqian Hong, Jie Zhang, Bo Zheng, Shaojun Zhu, Naimei Chen, Zhentao Zhu, Weihua Yang

**Affiliations:** ^1^School of Information Engineering, Huzhou University, Huzhou, China; ^2^Zhejiang Province Key Laboratory of Smart Management and Application of Modern Agricultural Resources, Huzhou University, Huzhou, China; ^3^Shenzhen Eye Hospital, Jinan University, Shenzhen, China; ^4^Advanced Ophthalmology Laboratory, Brightview Medical Technologies (Nanjing) Co., Ltd., Nanjing, China; ^5^Department of Ophthalmology, Huaian Hospital of Huaian City, Huaian, China

**Keywords:** dry and wet macular degeneration classification models, intelligent assisted diagnosis, deep learning, ConvNeXT model, artificial intelligence

## Abstract

**Purpose:**

To assess the value of an automated classification model for dry and wet macular degeneration based on the ConvNeXT model.

**Methods:**

A total of 672 fundus images of normal, dry, and wet macular degeneration were collected from the Affiliated Eye Hospital of Nanjing Medical University and the fundus images of dry macular degeneration were expanded. The ConvNeXT three-category model was trained on the original and expanded datasets, and compared to the results of the VGG16, ResNet18, ResNet50, EfficientNetB7, and RegNet three-category models. A total of 289 fundus images were used to test the models, and the classification results of the models on different datasets were compared. The main evaluation indicators were sensitivity, specificity, F1-score, area under the curve (AUC), accuracy, and kappa.

**Results:**

Using 289 fundus images, three-category models trained on the original and expanded datasets were assessed. The ConvNeXT model trained on the expanded dataset was the most effective, with a diagnostic accuracy of 96.89%, kappa value of 94.99%, and high diagnostic consistency. The sensitivity, specificity, F1-score, and AUC values for normal fundus images were 100.00, 99.41, 99.59, and 99.80%, respectively. The sensitivity, specificity, F1-score, and AUC values for dry macular degeneration diagnosis were 87.50, 98.76, 90.32, and 97.10%, respectively. The sensitivity, specificity, F1-score, and AUC values for wet macular degeneration diagnosis were 97.52, 97.02, 96.72, and 99.10%, respectively.

**Conclusion:**

The ConvNeXT-based category model for dry and wet macular degeneration automatically identified dry and wet macular degeneration, aiding rapid, and accurate clinical diagnosis.

## Introduction

Macular degeneration, a neurodegenerative diseases and blinding eye disease, is the leading cause of irreversible central vision loss in developed countries and is expected to affect 288 million people worldwide by 2040 (Wong et al., 2014; Rozing et al., 2020). Macular degeneration can be divided into dry macular degeneration and wet macular degeneration. They differ in terms of prevalence, clinical symptoms, speed of onset, and treatment, and the distinction between the two is crucial in clinical diagnosis (Gelinas et al., 2022; Sarkar et al., 2022; Shen et al., 2022). The traditional diagnosis of macular degeneration relies on stereo-ophthalmoscopy, fundus photography, and optical coherence tomography retinal imaging, which are inefficient due to the high demand for medical resources for diagnosis; and some patients are unable to obtain a timely diagnosis, resulting in a delay in treatment (Qu et al., 2020; Rubner et al., 2022). Therefore, the design of an automatic classification model of dry and wet macular degeneration will aid in large-scale screening of dry and wet macular degeneration, improve the efficiency of disease diagnosis, compensate for the shortage of primary medical resource distribution, and facilitate the early detection, diagnosis, and treatment of the disease.

Recently, artificial intelligence technology has shown broad application prospects in ophthalmology. Current research hotspots are based on the automated analysis of fundus images, including segmentation of anatomical structures, detection, and classification of lesion sites, diagnosis of ocular diseases, and image quality assessment (Zheng et al., 2021a,b; Zhu et al., 2022), involving common eye diseases, such as diabetic retinopathy (Bora et al., 2021; Sungheetha and Sharma, 2021; Wan et al., 2021), glaucoma (Mahum et al., 2021; Ran et al., 2021), age-related macular degeneration (AMD) (Moraes et al., 2021; Thomas et al., 2021), and cataracts (Khan et al., 2021; Gutierrez et al., 2022). At present, artificial intelligence technology has made breakthrough progress in the field of macular degeneration, and its application in research is mainly focused on macular degeneration identification and macular degeneration severity grading. Celebi et al. (2022) achieved automatic detection of macular degeneration with an accuracy of 96.39%. Serener and Serte (2019) classified macular degeneration with 94% accuracy based on the ResNet18 model. Abdullahi et al. (2022) achieved the classification of macular degeneration based on ResNet50 with an accuracy of 96.56%. Few studies have classified dry and wet macular degeneration. Priya and Aruna (2014) used machine learning methods, such as probabilistic neural networks, to extract retinal features and classify dry and wet macular degeneration with a maximum accuracy, sensitivity, and specificity of 96, 96.96, and 94.11%, respectively, while Chen et al. (2021) used a multimodal deep learning framework to automate the detection of dry and wet macular degeneration with a maximum accuracy, sensitivity, and specificity of 90.65, 68.92, and 98.53%, respectively. In some of these studies, the evaluation indicators of the models were not sufficiently comprehensive, the sensitivity and specificity values of some models varied widely, and some models studied only dichotomous categories, with limited practical application.

Based on clinically collected normal, dry, and wet macular degeneration fundus images, this study trained the ConvNeXT (Liu et al., 2022) three-category model on the original and expanded datasets and compared the results with the VGG16 (Simonyan and Zisserman, 2014), ResNet18 (He et al., 2016), ResNet50 (Khan et al., 2018), EfficientNetB7 (Tan and Le, 2019), and RegNet (Radosavovic et al., 2020) three-category models. It is expected that, in this study, a suitable model can be found to assist AMD diagnosis.

## Materials and methods

### Data source

The macular fundus image dataset used in this study was obtained from the Eye Hospital of Nanjing Medical University as JPG RGB color fundus images with good image quality. As factors, such as sex and age, did not affect the results of this study, and to prevent leakage of patients’ personal information, all images were desensitized before delivery and did not contain any private patient information. The partner hospital provided the images along with the true diagnostic result for each image, which was used as the result for the expert diagnostic group. All images were reviewed by two ophthalmologists. If the diagnosis was consistent, the final diagnosis was made; if the diagnosis was inconsistent, the final diagnosis was made by a third ophthalmologist. The fundus images provided by the partner hospital contain only one of the following: normal, dry macular degeneration, or wet macular degeneration; meaning that the diagnosis is unique.

**FIGURE 1:**
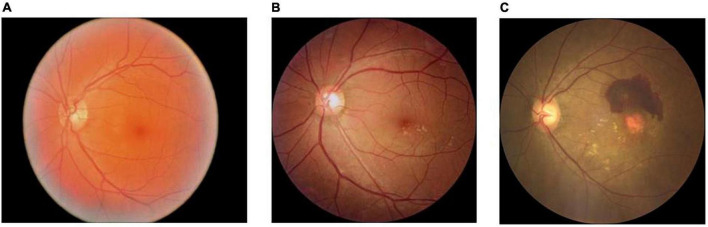
Normal, dry, and wet macular fundus images. **(A)** The normal fundus image; **(B)** the dry macular degeneration fundus image; and **(C)** the wet macular degeneration fundus image.

In this study, the original dataset consisted of 672 fundus images, which were first divided into a training set and a validation set according to 9:1. The training set consisted of 604 images, including 252 normal fundus images, 100 dry macular degeneration fundus images, and 252 wet macular degeneration fundus images; the validation set consisted of 68 images, including 28 normal fundus images, 12 dry macular degeneration fundus images, and 28 wet macular degeneration fundus images. In this study, 289 fundus images obtained from the clinic were used as the testing set, and the experts gave a diagnosis of 120 normal fundus, 48 dry macular degeneration, and 121 wet macular degeneration. The number of original data sets is limited and the distribution is not uniform. The number of images per category in each dataset is shown in Table 1. Fundus images of normal, dry, and wet macular degeneration are shown in Figure 1.

**TABLE 1:** Original data set division.

	Training	Validation	Test
Normal	252	28	120
Dry	100	12	48
Wet	252	28	121
Total	604	68	289

### Data augmentation

**FIGURE 2:**
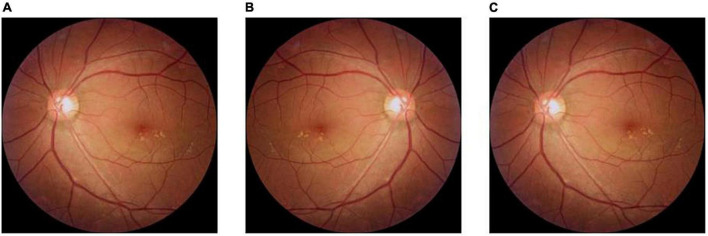
Method of expanding fundus images in dry macular degeneration. **(A)** The original image; **(B)** the image after horizontal flip; and **(C)** the image after a 3° counterclockwise rotation.

In this study, the amount of labeled data in the fundus image dataset is limited and the number of images in the three categories is unbalanced, which may lead to under-fitting in the category with a small amount of data and over-fitting in the category with a large amount of data during model training. Although deep learning has shown great potential in the field of smart healthcare, most of the existing methods can only handle data with high quality, sufficient data volume, balanced categories, and the same source. In order to improve the applicability of the model in helping the initial classification and diagnosis of macular degeneration, the fundus images of dry macular degeneration with the least amount of data in the training set were expanded. First, the original 100 dry fundus images were flipped horizontally, and then half of the original images were rotated 3° counterclockwise to obtain 250 dry fundus images, which balanced the number of different kinds of experimental data and ensured the generalization ability of the model. The original image, the image after horizontal rotation, and the image after 3° counterclockwise rotation are shown in Figure 2.

The data in the dataset were enhanced to improve the robustness and accuracy of the model. The data enhancement methods are as follows: cropping the images by setting a random aspect ratio, where the random size is set to 0.08–1 times the original image, and the random aspect ratio is set to 0.75–1.33 times of the original image; and horizontally flipping the image at random with a 50% chance.

### Model training

In this study, the classification models were trained on the original dataset and the expanded dataset, respectively, and the models were tested using the same test dataset. The number of images per category in the original and expanded datasets is shown in Table 2. The Adam optimization algorithm was used in this study, and after 40 data iterations, the initial value of the learning rate was set to 0.0005. The dynamic adjustment strategy was to first calculate the multiplication factor based on the number of training iterations, and then multiply it with the initial learning rate. The initial parameters were loaded with those obtained from training ConvNeXT-T on ImageNet-1K. Meanwhile, the VGG16, ResNet18, ResNet50, EfficientNetB7, and RegNet models were selected to compare the classification results, all of which contain convolutional layers, pooling layers, and fully connected layers.

**TABLE 2:** Comparison of original and expanded datasets.

	Original data		Expanded data	
	Training	Validation	Training	Validation
Normal	252	28	252	28
Dry	100	12	250	12
Wet	252	28	252	28
Total	672		822	

**FIGURE 3:**
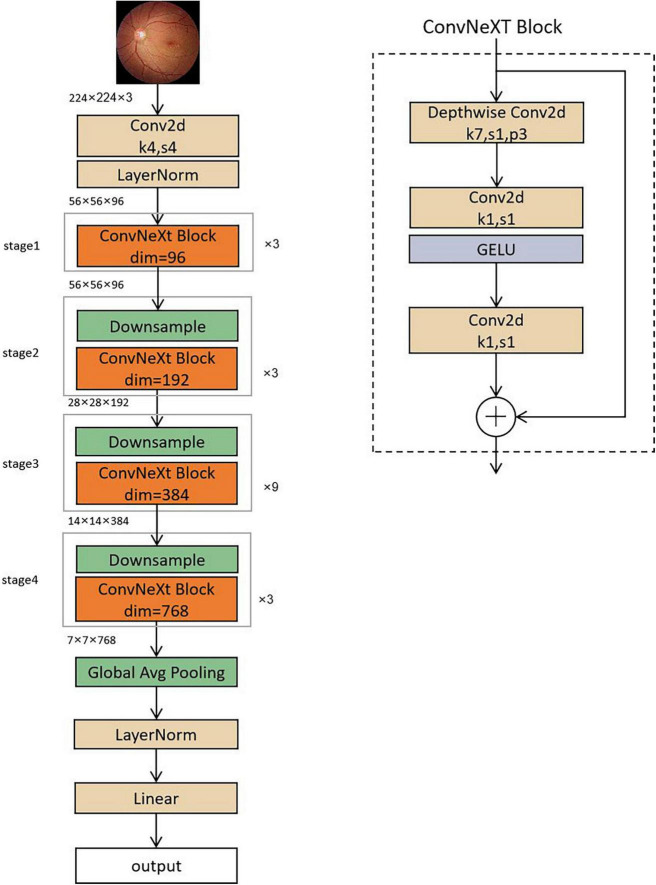
Structure of the ConvNeXT model.

The ConvNeXT model has an input image size of 224 × 224, which is down sampled by a convolutional layer. The image size is reduced to 56 × 56, passing through four stages in turn, each consisting of a series of ConvNeXT blocks, and then passed through a global average pooling layer, normalization, and a full connection layer to obtain the final classification result. The structure of the ConvNeXT model is shown in Figure 3. The input feature matrix of the ConvNeXT block enters the residual block with two branches. The straight branch first goes through a depth-separable convolution, then through two convolutions output the feature matrix, which is added to the original input branch to obtain the final feature matrix.

### Statistical analysis

Statistical analysis was performed using IBM SPSS Statistics for Windows version 25.0 (IBM Corp., Armonk, NY, USA). The count data are expressed as the number of images and percentages. The sensitivity, specificity, F1-score, and area under the curve (AUC) of the macular degeneration intelligent aid diagnostic model for the diagnosis of normal, dry macular degeneration, and wet macular degeneration were calculated, receiver operating characteristic curves (ROCs) were plotted, and the consistency of the diagnostic results of the expert diagnostic group and the model were assessed using the Kappa test. The results of the expert diagnostic group were used as the clinical diagnostic criteria, and kappa values of 0.61–0.80 were considered as significant agreement, >0.80 was considered high agreement; ROC curves were used to analyze the diagnostic performance of different models; and AUC values of 0.50–0.70 were considered as low diagnostic values, 0.70–0.85 were considered average, >0.85 was considered good diagnostic value.

## Results

In this study, 289 clinical fundus images were used as test sets to assess the three-category models of dry and wet macular degenerations. The expert diagnostic group diagnosed 120 patients with normal fundus, 48 with dry macular degeneration, and 121 with wet macular degeneration. The results of the ConvNeXT three-category model on the original dataset were as follows: 120 normal fundus, 34 dry macular degeneration, and 118 wet macular degeneration. The results of the ConvNeXT three-category model on the expanded dataset were as follows: 120 normal fundus, 42 dry macular degeneration, and 118 wet macular degeneration. The diagnostic results of the model are presented in Tables 2, 3.

**TABLE 3:** Diagnostic results of the ConvNeXT model on the original dataset.

Clinical	ConvNeXT diagnoses			
	Normal	Dry	Wet	Total
Normal	117	3	0	120
Dry	1	45	2	48
Wet	0	19	102	121
Total	118	67	104	289

**FIGURE 4:**
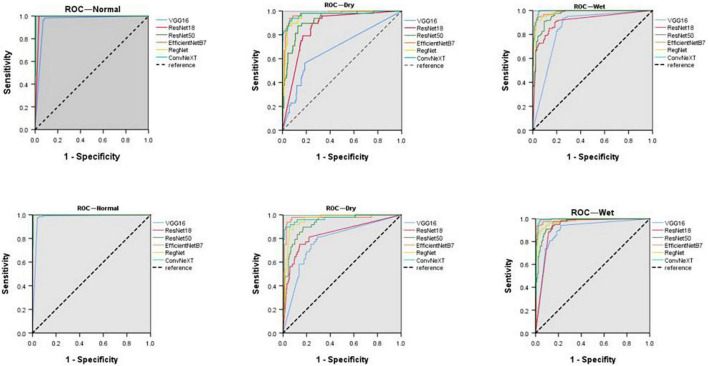
Receiver operating characteristic curves (ROCs) of normal fundus, dry, and wet macular degeneration on the original and extended data sets for six models. ROC, receiver operating characteristic curve.

**FIGURE 5:**
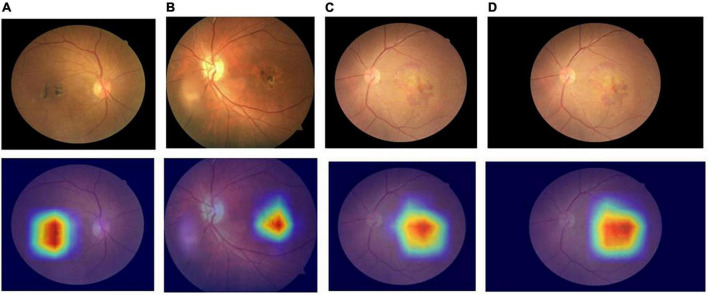
Heap maps of ConvNeXT model for dry and wet macular degeneration. Panels **(A,B)** the heap maps of dry macular degeneration; panels **(C,D)** the heap maps of wet macular degeneration.

Comparing the diagnostic results of the three-category models VGG16, ResNet18, ResNet50, EfficientNetB7, RegNet, and ConvNeXT on the original dataset, the EfficientNetB7 model had the best results, followed by the ConvNeXT model. After data expansion, the ConvNeXT model achieved the best results, surpassing the EfficientNetB7 model. On the expanded dataset, the ConvNeXT accuracy was 96.89%, and the kappa value was 94.99%. The sensitivity, specificity, F1-score, and AUC values of the model for the diagnosis of normal fundus images were 100.00, 99.41, 99.59, and 99.80%, respectively. Regarding the diagnosis of dry macular degeneration, the sensitivity, specificity, F1-score, and AUC values were 87.50, 98.76, 90.32, and 97.10%, respectively. For the diagnosis of wet macular degeneration, the sensitivity, specificity, F1-score, and AUC values were 97.52, 97.02, 96.72, and 99.10%, respectively. The evaluation indicators for the diagnostic results of each model are listed in Tables 4, 5. The ROC curves are shown in Figure 4. The heap maps of ConvNeXT model are shown in Figure 5.

**TABLE 4:** Diagnostic results of the ConvNeXT model on the expanded dataset.

Clinical	ConvNeXT diagnoses			
	Normal	Dry	Wet	Total
Normal	120	0	0	120
Dry	1	42	5	48
Wet	0	3	118	121
Total	121	45	123	289

**TABLE 5:** Test results of different models trained on the original dataset.

Model	Evaluation indicators	Normal (%)	Dry (%)	Wet (%)
VGG16	Sensitivity	97.50	50.00	74.38
	Specificity	95.27	86.31	89.88
	F1-score	95.51	45.71	78.95
	AUC	0.955	0.683	0.854
	95% CI	0.929–0.982	0.594–0.771	0.809–0.899
	Kappa		68.28	
	Accuracy		80.13	
	Model size (MB)		747	
	Model parameters (Million)		138	
ResNet18	Sensitivity	**100.00**	75.00	66.94
	Specificity	96.45	83.40	96.43
	F1-score	97.56	58.06	77.88
	AUC	0.982	0.861	0.912
	95% CI	0.966–0.999	0.810–0.911	0.875–0.949
	Kappa		72.29	
	Accuracy		82.01	
	Model size (MB)		137	
	Model parameters (Million)		12	
ResNet50	Sensitivity	**100.00**	81.25	77.69
	Specificity	**99.41**	88.80	95.24
	F1-score	**99.59**	68.42	84.30
	AUC	0.999	0.922	0.963
	95% CI	0.997–1	0.885–0.960	0.944–0.981
	Kappa		80.55	
	Accuracy		87.54	
	Model size (MB)		384	
	Model parameters (Million)		26	
EfficientNet-B7	Sensitivity	**100.00**	89.58	90.08
	Specificity	**99.41**	95.02	97.62
	F1-score	**99.59**	83.50	93.16
	AUC	**1**	0.970	0.987
	95% CI	**0.998–1**	0.946–0.993	0.977–0.996
	Kappa		90.68	
	Accuracy		94.11	
	Model size (MB)		1,919	
	Model parameters (Million)		66	
RegNet	Sensitivity	**100.00**	87.50	87.60
	Specificity	**99.41**	93.78	97.02
	F1-score	**99.59**	80.00	91.38
	AUC	0.999	0.963	0.984
	95% CI	0.998–1	0.946–0.986	0.974–0.994
	Kappa		88.51	
	Accuracy		92.73	
	Model size (MB)		221	
	Model parameters (Million)		23	
ConvNeXT	Sensitivity	97.50	**93.75**	84.30
	Specificity	**99.41**	**90.87**	**98.81**
	F1-score	98.32	78.26	90.67
	AUC	0.997	**0.972**	**0.994**
	95% CI	0.991–1	**0.939–1**	**0.988–0.999**
	Kappa		**92.39**	
	Accuracy		91.35	
	Model size (MB)		**106**	
	Model parameters (Million)		28	

Bold indicates the best results for each type of fundus image on different evaluation criteria after diagnosis by the model.

## Discussion

Macular degeneration is a relatively common and irreversible blinding fundus disease worldwide. Studies have shown that, as life expectancy increases, macular degeneration is one of the leading causes of vision loss in the elderly (Gong et al., 2021). However, in less economically developed countries and regions, the uneven distribution of medical resources and the long training period for professional ophthalmologists prevents a large number of AMD patients from being diagnosed and treated timeously. If manual diagnostic screening is used, it takes a considerable amount of time and human training costs. The application of an automated diagnostic system can provide a good platform for regular screening, effectively alleviating the medical needs of a large number of patients, and is vital to reducing the rate of blindness or impaired visual function due to macular degeneration (Cai et al., 2020). Thus, this study may assist ophthalmologists in the initial classification and diagnosis of macular degeneration by training an automatic classification model to improve diagnostic efficiency and benefit patients.

Compared with other classification models, the ConvNeXT-based classification model for dry and wet macular degeneration obtained excellent results. It borrowed ideas from Swin transformer and used training strategies such as depth-separable convolution, image down sampling, and increasing convolution kernel to extract a larger number of features at a higher semantic levels from fundus images and to reduce the computational effort, accelerate feature extraction, and stabilize model training, thus enhancing the expressive power of the model (Zhang et al., 2022). In addition, this study found that the EfficientNetB7 model gave the best results on the original dataset, the ConvNeXT model gave the best results on the expanded dataset, and the results of the EfficientNetB7 model were differed only slightly from those of the ConvNeXT model. Since EfficientNetB7 is a traditional convolutional neural network, the accuracy of the model is improved by scaling network dimensions such as width and depth. ConvNeXT is a new convolution based architecture with less inductive bias, introducing the inherent advantages of transformer. ConvNeXT can often perform slightly better than EfficientNetB7 when the amount of data increases. Meanwhile, the EfficientNetB7 model was nearly 20 times larger than ConvNeXT, and the number of parameters in the EfficientNetB7 model was nearly three times larger than that of ConvNeXT. Therefore, the ConvNeXT model is more suitable for practical medical diagnosis. In addition, some of the model results need to be improved, mainly because the fundus images themselves are more complex, and these models have a simpler structure compared to ConvNeXT and EfficientNetB7, and do not extract the image features sufficiently.

As shown in Tables 5, 6, the evaluation indices of normal fundus images are generally higher than the corresponding indices of dry and wet macular degeneration fundus images. The main reason for this is that the differences between the normal fundus images and macular degeneration fundus images are large and easy to distinguish. Even professional ophthalmologists have difficulty making accurate diagnoses of all cases through fundus images alone, so the diagnostic results of the model are slightly poor. In future, we will consider combining features extracted by deep learning with manually selected features to extract more comprehensive image features and further improve the sensitivity and specificity of the model. Simultaneously, the fusion of manually selected features can increase the interpretability of the features.

**TABLE 6:** Test results of different models trained on the expanded dataset.

Model	Evaluation indicators	Normal (%)	Dry (%)	Wet (%)
VGG16	Sensitivity	97.50	54.17	74.38
	Specificity	95.86	86.31	90.48
	F1-score	95.90	48.60	79.30
	AUC	0.974	0.780	0.899
	95% CI	0.954–0.994	0.706–0.854	0.860–0.937
	Kappa		69.45	
	Accuracy		80.62	
	Model size (MB)		747	
	Model parameters (Million)		138	
ResNet18	Sensitivity	**100.00**	41.67	92.56
	Specificity	97.63	96.27	85.71
	F1-score	98.36	51.95	87.16
	AUC	0.997	0.836	0.925
	95% CI	0.990–1	0.764–0.908	0.893–0.958
	Kappa		78.95	
	Accuracy		87.20	
	Model size (MB)		137	
	Model parameters (Million)		12	
ResNet50	Sensitivity	99.17	62.50	90.91
	Specificity	99.41	95.44	89.29
	F1-score	99.17	67.42	88.35
	AUC	0.998	0.928	0.965
	95% CI	0.995–1	0.892–0.964	0.947–0.983
	Kappa		83.22	
	Accuracy		89.62	
	Model size (MB)	384		
	Model parameters (Million)		26	
EfficientNet-B7	Sensitivity	**100.00**	**93.75**	92.56
	Specificity	**99.41**	96.27	**98.81**
	F1-score	**99.59**	88.24	95.32
	AUC	**1**	0.968	0.983
	95% CI	**0.999–1**	0.936–1	0.968–0.998
	Kappa		93.41	
	Accuracy		95.84	
	Model size (MB)		1,919	
	Model parameters (Million)		66	
RegNet	Sensitivity	**100.00**	85.42	89.26
	Specificity	**99.41**	94.61	96.43
	F1-score	**99.59**	80.39	91.91
	AUC	**1**	0.960	0.981
	95% CI	**0.999–1**	0.938–0.982	0.970–0.993
	Kappa		89.01	
	Accuracy		93.08	
	Model size (MB)		221	
	Model parameters (Million)		23	
ConvNeXT	Sensitivity	**100.00**	87.50	**97.52**
	Specificity	**99.41**	**98.76**	97.02
	F1-score	**99.59**	**90.32**	**96.72**
	AUC	0.998	0.971	**0.991**
	95% CI	0.994–1	**0.941–1**	**0.982–1**
	Kappa		**94.99**	
	Accuracy		**96.89**	
	Model size (MB)		**106**	
	Model parameters (Million)		28	

Bold indicates the best results for each type of fundus image on different evaluation criteria after diagnosis by the model.

In this study, the original data were small and unevenly distributed, and data expansion was used to equalize the number of fundus images for each category. Comparing Tables 5, 6, it can be seen that the evaluation metrics of all models improved after data expansion, with ResNet18 improving the most, with a 5.19% increase in accuracy and a 6.66% increase in kappa value; VGG16 improved the least, with a 0.49% increase in accuracy and a 1.17% increase in kappa value. Thus, data expansion allows the model to learn features effectively for each category of images rather than focusing on categories with a larger number of samples; thus, improving the expressiveness of the model. The modern field of intelligent medical diagnosis generally has problems, such as a low amount of annotated data and data imbalance. This research team will gather more training data, while focusing on the progress of research on training models on small sample datasets, with the intention of conducting further research in the future.

Priya and Aruna (2014) used a machine learning approach to extract retinal image features for classification, with the support vector machine (SVM) classifier achieving a maximum accuracy of 96%. Chen et al. (2021) used a multimodal deep-learning framework to automatically classify macular degeneration with a maximum accuracy of 90.65%. Traditional machine learning algorithms exhibit poor generalization performance and are prone to over-fitting problems. The deep learning effect was more prominent when the amount of data increased. Multimodal deep learning frameworks require datasets containing diverse forms of color fundus images, optical coherence tomography, and fundus autofluorescence images, which are difficult to acquire and can add to the burden of human and medical resources. In this study, the ConvNeXT model was used to classify dry and wet macular degeneration, and only color fundus images were required. It was widely used in clinical applications, and the data were easy to obtain, with an accuracy of 96.89%, and demonstrated superior results. Simultaneously, the ConvNeXT model has fewer parameters and requires less memory than other models, making it easier to apply to end devices.

## Conclusion

In this study, automatic classification of normal, dry, and wet macular degeneration was implemented based on the ConvNeXT model. Twelve models were trained on the original and expanded datasets. Results showed that the ConvNeXT model trained on the expanded dataset obtained high sensitivity, specificity, and accuracy and could be used to develop an automatic classification system for dry and wet macular degeneration. This automatic classification system may provide a good platform for regular screening in primary care and could help address the problem of many patients in less economically developed areas with fewer medical resources.

## Data availability statement

The original contributions presented in this study are included in this article/supplementary material, further inquiries can be directed to the corresponding authors.

## Author contributions

MW and YL wrote the manuscript. BZ planned the experiments and the manuscript. SZ guided the experiments. YL and BZ trained the model. WY and MW reviewed the manuscript. XH, JZ, and ZZ collected and labeled the data. All authors issued final approval for the version to be submitted.
